# Natural motion trajectory enhances the coding of speed in primate extrastriate cortex

**DOI:** 10.1038/srep19739

**Published:** 2016-01-27

**Authors:** Amanda J. Davies, Tristan A. Chaplin, Marcello G. P. Rosa, Hsin-Hao Yu

**Affiliations:** 1 Department of Physiology, Monash University, Clayton, Victoria 3800, Australia; 2 Monash Vision Group, Monash University, Clayton, Victoria 3800, Australia; 3 Australian Research Council Centre of Excellence for Integrative Brain Function.

## Abstract

The ability to estimate the speed of an object irrespective of size or texture is a crucial function of the visual system. However, previous studies have suggested that the neuronal coding of speed in the middle temporal area (MT, a key cortical area for motion analysis in primates) is ambiguous, with most neurons changing their speed tuning depending on the spatial frequency (SF) of a visual pattern. Here we demonstrate that the ability of MT neurons to encode speed is markedly improved when stimuli follow a trajectory across the visual field, prior to entering their receptive fields. We also show that this effect is much less marked in the primary visual area. These results indicate that MT neurons build up on computations performed at earlier levels of the visual system to provide accurate coding of speed in natural situations, and provide additional evidence that nonlinear pooling underlie motion processing.

Neurons in the middle temporal area (MT) are regarded as key components of the brain network that processes visual motion^1^, but previous studies have questioned the extent to which their activity can yield reliable information about speed. Using a classical visual physiology paradigm, these studies have used local motion patterns (drifting sinusoidal gratings), positioned over the receptive fields of MT neurons, to characterize their tuning to speed. Because speed can be derived from the ratio between the temporal frequency (TF) and spatial frequency (SF) of a grating, and given that these two variables can be manipulated independently, this paradigm can be used to determine whether or not a neuron is speed-tuned. Specifically, for a truly speed-tuned neuron, the optimal stimulus TF of a grating is expected to increase with its SF, so the ratio between these variables remains constant^2^. However, with the exception of one study^3^, the results of these experiments have indicated that most MT neurons, like those in earlier stations of the visual system, are tuned to TF, not speed^4^,^5^,^6^.

There are, however, reasons to question whether earlier studies have provided a full picture of the ability of MT neurons to signal speed. The motion signals in drifting gratings originate from the temporal modulation of the spatial phases of localized carrier waves, but the stimulus envelopes remain stationary over the same retinal locations. With rare exceptions^7^, this combination of features is not found in natural images, and therefore it can be argued that the primate brain may not have evolved to optimally evaluate speed in such situations.

That the behaviour of motion processing neurons can be characterized with drifting gratings has been justified, on theoretical grounds, by the fact that the images of objects that travel in straight trajectories are represented in Fourier space as oriented lines or planes, and can therefore be captured by linear filters^8^,^9^. This being the case, the responses of neurons that process motion using linear summation of signals could be adequately characterized using drifting gratings. However, the summation linearity assumption has already been challenged by the demonstration that MT neurons show greater evidence of speed-tuning when stimulated with two superimposed gratings of different SFs^4^, a result that established that the Fourier components of a moving pattern can interact nonlinearly to alter information processing in the primate brain.

Translation of an object across visual space affects the exact combination of spatial and temporal frequencies to which the visual system is exposed, thus also creating opportunity for nonlinear interactions in neural processing. However, it has not yet been tested whether this type of moving pattern elicits a greater degree of SF-invariant speed-tuning in visual cortex. To test if this is the case, in the present study we compared the responses of MT neurons to drifting Gabor patches (sinusoidal gratings weighted by a Gaussian envelope) and Gabor patches that moved across the receptive field, from a different retinal location. The use of the latter type of stimulus allowed us to incorporate motion trajectory, an important feature of natural motion signals, while retaining the ability to vary SF and TF in a precise manner. With this method, we show that stimuli that move across the receptive field of a neuron are processed differently from locally drifting stimuli, and that only the first elicit robust speed-tuning in a large number of MT cells.

## Results

**Figure 1:**
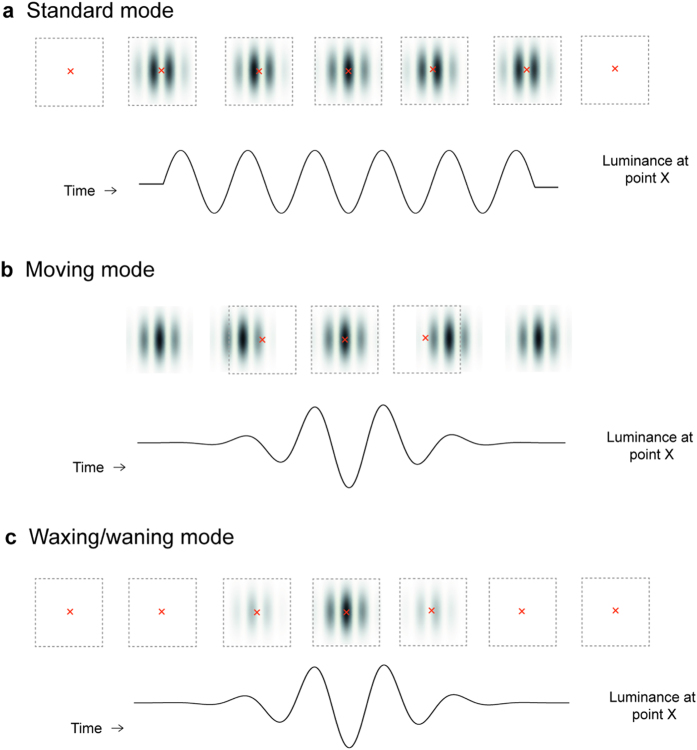
The three modes of stimulus presentation. For each panel, the top row illustrates the spatial relationship between the Gabor patch and the receptive field (indicated by the dotted squares) at several time points. The bottom row represents the evolution of stimulus luminance at the centre of the receptive field (marked by a red cross). There was a 1 s stationary period for Gabor patches in the standard and the moving mode (see *Methods*), which is not shown in this figure for simplicity.

We characterized the response properties of MT and V1 neurons by presenting high-contrast Gabor patches to their receptive fields, according to three stimulus presentation modes (see Fig. 1 and Supplementary Video 1).

The *standard mode* replicated the typical way in which drifting gratings were presented in earlier experiments that examined speed-tuning in visual cortex: the sinusoidal carrier of a Gabor patch drifted within a stationary envelope centred on the cell’s receptive field, for a fixed time interval (Fig. 1a). However, the use of a Gabor patch instead of a traditional grating with sharp boundaries eliminates the possible confound represented by richer combinations of spatial frequencies at the edges of the stimulus. In the *moving mode*, a non-drifting Gabor patch appeared well outside the neuron’s receptive field, and then moved in a straight trajectory, across the receptive field (Fig. 1b). The *waxing/waning mode* was designed to mimic the effect of viewing a moving stimulus through an aperture corresponding to the receptive field: the Gabor patch drifted within a stationary envelope, as in the standard mode, but the global contrast of the Gabor pattern was temporally modulated in such a way that the time course of the modulation at the centre of the receptive field was matched to that of the corresponding stimulus in the moving mode (Fig. 1c). In all three modes, the sinusoidal components of the Gabor pattern moved at a constant speed at each point in the cell’s receptive field, but only in the moving mode did the envelope of the pattern move across the visual field. In all tests, the diameter of the Gabor patch was set to be 10% larger than that of the receptive field (carefully mapped with moving bars and small gratings), to further reduce the possibility of boundary effects, and the direction of motion was set to the neuron’s optimal, determined quantitatively.

**Figure 2:**
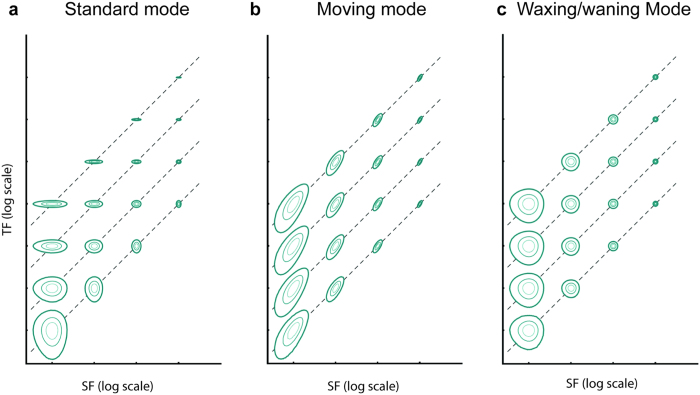
Schematic representations of the three stimulus modes in the Fourier space. The energy distributions of the Gabor patches displayed in the three modes are plotted in log-log scale. In this representation, SF and TF combinations that correspond to the same speeds (iso-speed lines, indicated by dashed lines in the figure) are tilted at 45° angles. Iso-speed lines closer to the x-axis represent slower speeds. In (**b**), the contours represent energy distributions of Gabor patterns passing through a fixed receptive field, whose envelope was modelled as a Gaussian function.

Figure 2 schematically illustrates how the Fourier space was sampled by the three stimulus modes. In the standard mode, the Fourier energy for each combination of spatial and temporal frequency corresponds to a bivariate Gaussian distribution with major and minor axes parallel to the spatial frequency (SF) and temporal frequency (TF) axes. The SF bandwidth (determined by the size of the Gabor patch) and the TF bandwidth (determined by the time during which the carrier wave drifted) were constant in all conditions, but as Fig. 2 is plotted in logarithmic scale, the Gaussian distributions appear to change their width and height as SF and TF increase. In the moving mode, the Gaussian distributions are tilted 45° in logarithmic scale, parallel to the iso-speed lines (dashed lines). The TF bandwidths varied with speed because the time interval during which the Gabor patch remained within the receptive field varied, but they appear constant in logarithmic scale. Finally, the energy of a Gabor patch in the waxing/waning mode closely matched to that in the moving mode, but was not tilted at a 45° angle, as in the moving mode. The TF bandwidth varied with speed, due to the variation of the duration of the stimulus.

### Single-neuron response patterns

We characterized the response properties of 110 neurons confirmed to be in area MT by histological reconstruction^10^. The receptive fields were in the peripheral visual field, between 10° and 30^o^ eccentricity, and encompassed both the upper and lower contralateral quadrants. The diameters of the receptive fields were in the range of 5° to 15° (median value 10.2°).

**Figure 3:**
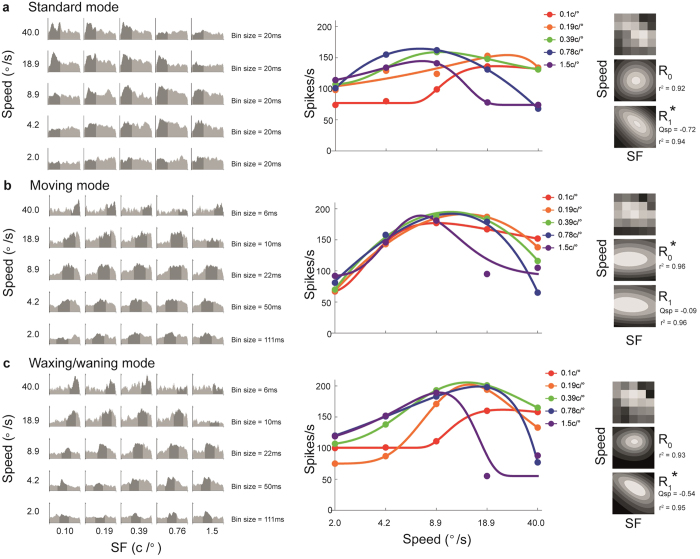
Data from a MT neuron that was speed-tuned only in the moving mode. Rows show responses to the (**a**) standard, (**b**) moving and (**c**) waxing/waning modes. For each panel, the first column shows the responses to each SF-speed combination as a peri-stimulus time histogram (PSTH). In these histograms, bin sizes (indicated at the right hand side of each row) were adjusted to represent equal distances travelled by the stimulus in the moving mode. The y-axis represents the response-rate calculated within the bins; scale is the same for all histograms, with the maximal value of 266Hz. The shaded regions represent the time intervals during which the response rates shown in the second and the third column were calculated. The second column displays speed-tuning curves obtained using Gabor patches of different SF. The circles represent the measured response rates, and the curves are fitted skewed log-Gaussian functions. The third column illustrates tuning surfaces describing the responses to the 5 × 5 matrices of conditions (top) and the surfaces were fitted to two models, *R*_0_ (middle) and *R*_1_ (bottom). The models that provided the better fits, as determined by P(*R*_0_|*R*_0_, *R*_1_), are indicated by asterisks. The quality of fit (*r*^*2*^) and the estimated Q_sp_ are also provided.

The responses of a neuron are illustrated in Fig. 3 as peristimulus time histograms (PSTHs, left) and speed-tuning curves (centre) obtained with stimuli of different SFs. The various speed-tuning curves were converted into tuning surfaces, which were then fitted with two models (right): an SF-speed separable model (*R*_0_), which assumed that the SF and speed-tuning curves were independent of each other, and an SF-speed inseparable model (*R*_1_), in which the optimal speed could vary systematically with the stimulus SF. We used the Bayesian Information Criterion (BIC^11^) to calculate P(*R*_0_|*R*_0_, *R*_1_), the probability that *R*_*0*_ provided a better fit to the data over *R*_1_. For this neuron, P(*R*_0_|*R*_0_, *R*_1_) was lower than 0.001 for stimulation in the standard and waxing/waning modes, indicating that, for drifting gratings, it was necessary to use a model that incorporates a systematic relationship between the optimal speed and the SF of the stimulus. However, when the same neuron was tested with Gabor patches presented in the moving mode (second row), P(*R*_0_|*R*_0_, *R*_1_) was 0.81, indicating that the pattern of responses was better described by a model according to which the neuron’s optimal speed was not influenced by the SF of the stimulus.

**Figure 4:**
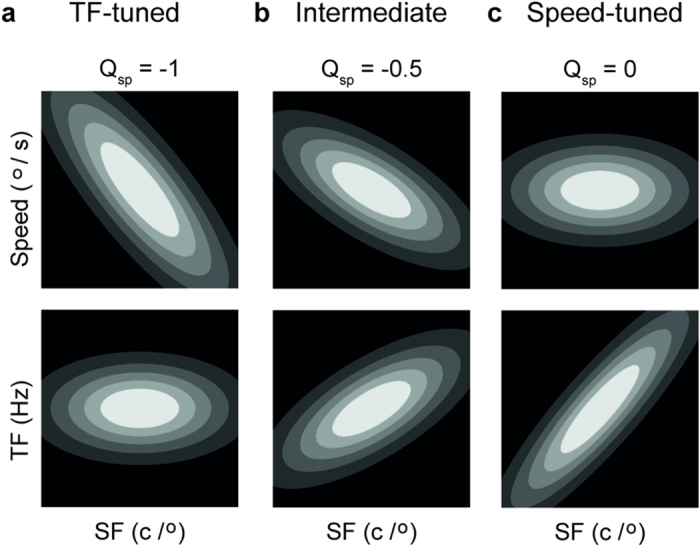
Schematic representations of the influence of parameter Q_sp_ in tuning surfaces fitted with model R_1_. The Q_sp_ parameter in the SF-speed inseparable model *R*_1_ allows the optimal speed to vary systematically with the SF of the stimulus. In this figure, the contours of the *R*_1_ model at 3 different levels of Q_sp_ are illustrated. In the upper row, contours are plotted in the SF-speed coordinates (in log-log scale). In the lower row, contours are converted into the SF-TF coordinates. (**a**) For Q_sp_ = −1, the optimal speed decreases with SF in a pattern that is consistent with perfect TF-tuning. (**b**) For −1 < Q_sp_ < 0, the optimal speed decreases with SF, but at a rate lower than expected from a TF-tuned neuron. (**c**) For Q_sp_ = 0, the optimal speed is independent with SF. This reflects a perfectly speed-tuned neuron.

The Q_sp_ parameter in R_1_ (see Fig. 4, and *Methods*) specifies the rate at which the optimal speed changes with the SF of the stimulus. This is analogous to the Q parameter used in previous studies, for quantifying the change in optimal TF with varying SF^4^,^5^,^6^. In the present convention, if Q_sp_ = 0, the optimal speed is independent of SF; in such a case R_1_ is equivalent to the SF-speed separable model, R_0_ (Fig. 4c). If Q_sp_ = −1, the optimal speed decreases with SF in a pattern that is consistent with perfect TF-tuning (Fig. 4a), whereas −1 < Q_sp_ < 0 (Fig. 4b) indicates that the optimal speed decreases with SF, but at a rate lower than expected from a TF-tuned cell. The Q_sp_ values estimated from the fitted models (third column in Fig. 3) were −0.72, −0.09 and −0.54 for the standard, moving and waxing/waning modes, respectively. The confidence intervals for Q_sp_ values obtained using the first and the third stimulus modes did not include 0, confirming that this neuron did not show SF-invariant speed-tuning when tested with drifting gratings.

**Figure 5:**
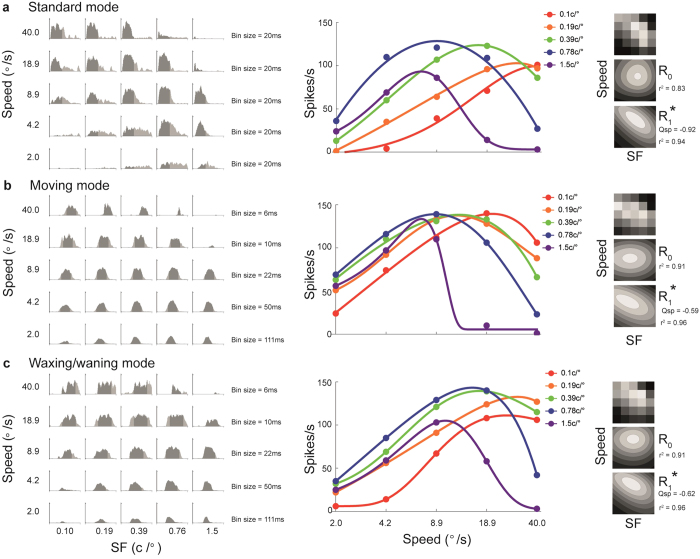
Data from a MT neuron that was not speed-tuned in all three modes. The data are presented in the same format as in Fig. 3. The y-axis in all PSTHs uses the same scale (maximum 192 Hz).

Our main finding is that approximately half of the MT neurons sampled showed clearer speed-tuning when stimulated by a moving Gabor patch (similar to the unit shown in Fig. 3). The other half had responses similar to the ones illustrated in Fig. 5, for which SF-invariant speed-tuning was not evident. For this cell, P(*R*_0_|*R*_0_, *R*_1_) was lower than 0.001 for stimulation in all three modes, showing that the SF-speed inseparable model provided a better description of its responses. In addition, the confidence intervals for the Q_sp_ values (−0.92, −0.59 and −0.62) obtained using the standard, moving, and waxing/waning modes did not include 0, but all included −1, further indicating that the responses of this neuron were better characterized as being tuned to the stimulus TF.

### Estimates of the number of speed-tuned neurons in MT depend on the stimulus mode

**Figure 6:**
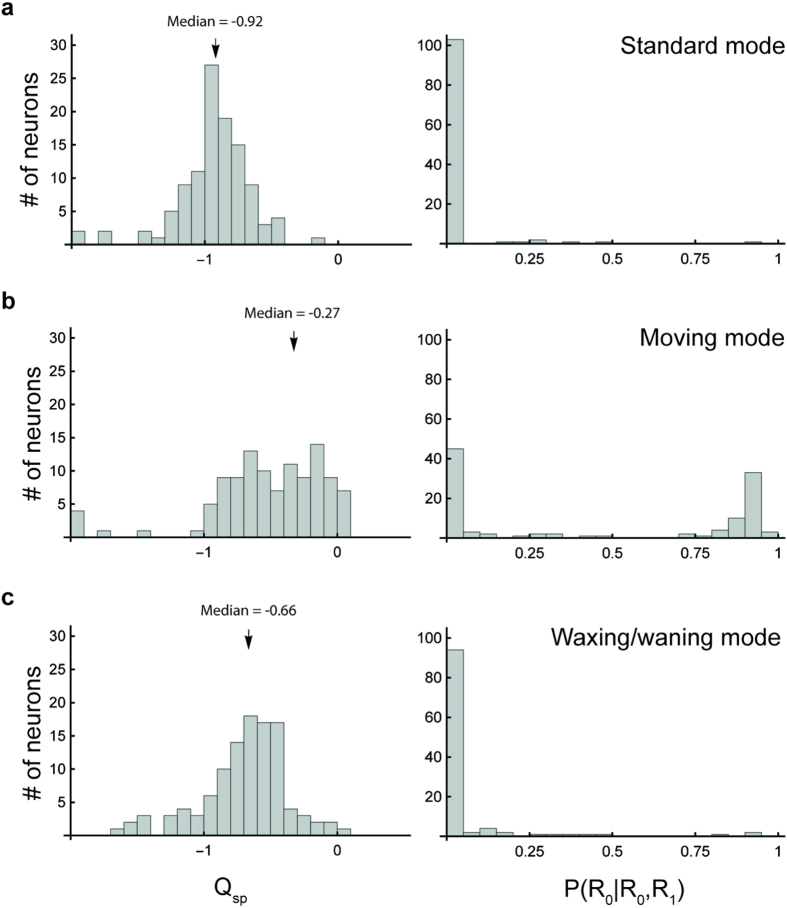
Distributions of Q_sp_ and P(*R*_0_|*R*_0_, *R*_1_) under the stimulation of the 3 modes in MT. (**a**) Histograms showing the distributions of Q_sp,_ The arrows indicate median values. (**b**) Histograms showing the distributions of P(*R*_0_|*R*_0_, *R*_1_).

In the left column of Fig. 6, we compare values of Q_sp_ that were obtained using the R_1_ (SF-speed inseparable) model to fit data obtained using the three stimulus modes. Consistent with previous studies^4^,^5^,^6^, these distributions were unimodal, and compatible with the notion that there is a continuum between speed-tuning and TF-tuning among MT neurons. Crucially, the distributions were shifted according to the mode of presentation of the stimulus. Median values of Q_sp_ were −0.92, −0.27 and −0.66 for the standard, moving and waxing/waning modes, respectively, indicating that the average dependency between SF and speed across the sample was highest in the standard mode, and lowest in the moving mode. The median values were significantly different from each other (Mann-Whitney Test; standard vs. moving: U = 642, p < 0.0001; standard vs. waxing/waning: U = 1024, p = 0.0001; waxing/waning vs. moving: U = 4647, p < 0.0001).

Estimating the number of speed-tuned neurons from a continuum of Q_sp_ depends on the choice of the criterion. As described above, we used a Bayesian method^11^ to decide if a simpler model (*R*_0_), which assumes speed-tuning, was more likely to provide a better description of the data, in comparison to the more complex model (*R*_1_). The distributions of P(*R*_0_|*R*_0_, *R*_1_), calculated using the BIC, are plotted in Fig. 6 (right column). They were unimodal and heavily skewed towards 0 for tests in the standard and waxing/waning modes. In marked contrast, the distribution for the moving mode was bimodal (Dip test, D = 0.101, p < 0.0001), with a clear increase in the number of neurons for which R_0_ was superior. Kolmogorov-Smirnov tests indicated that the distributions of P(*R*_0_|*R*_0_, *R*_1_) were significantly different between data obtained in the moving mode compared to the other two stimulus modes (standard vs. moving: D = 0.5636; p < 0.0001; waxing/waning vs. moving: D = 0.4527, p < 0.0001).

Using a threshold criterion P(*R*_0_|*R*_0_, *R*_1_) > 0.5, we found that only 1 cell (0.9%) would most likely be speed-tuned when tested with Gabor patches presented in the standard mode. That this estimate is even lower than that obtained by previous studies of speed tuning in MT (10–25%^4^,^5^,^6^) is likely related to our use of Gabor patches, which allow for a more precise control of the spatial frequencies within the receptive fields. Notably, this estimate increased dramatically to 53 (48.2%) when stimuli in the moving mode were used. For the waxing/waning mode stimulus, the corresponding number of cells was 3 (2.7%). There was no significant difference found between the proportions of speed-tuned cells revealed by the standard and waxing/waning modes (χ^2^ = 1.02, p = 0.3129), but these were statistically different from the result obtained using the moving mode (standard vs. moving: χ^2^ = 66.4, p < 0.0001; waxing/waning vs. moving: χ^2^ = 59.89, p < 0.0001). To confirm the reliability of the classification based on P(*R*_0_|*R*_0_, *R*_1_), we sought neurons for which the *R*_1_ model yielded Q_sp_ values between −0.25 and 0.25. This criterion was chosen with the heuristics that the fitted *R*_1_ surfaces with Q_sp_ values within this interval were often very similar to the fitted *R*_0_ surface. The numbers of speed-tuned cells according to this criterion were 1 (0.9%), 51 (46.4%), and 3 (2.7%), according to tests conducted in the standard, moving, and waxing/waning modes, respectively, thus confirming that our findings were not simply due to the method of classification. The first and the third values were, again, significantly different from those obtained using stimuli in the moving mode (standard vs. moving: χ^2^ = 62.96, p < 0.0001; waxing/waning vs. moving: χ^2^ = 50.69, p < 0.0001), but were not significantly different from each other (standard vs. waxing/waning: χ^2^ = 1.02, p = 0.3129). In summary, only tests using stimuli presented in the moving mode revealed a large population of speed-tuned cells in MT, irrespective of the classification criterion used.

### Many MT neurons only revealed speed-tuned responses to the moving mode stimulus

The population analyses described above prompt the question of whether there was a consistent pattern of change in speed-tuning classification, at the level of individual neurons. As summarized in Table 1, we found that 58 neurons (52.7% of the sample) did not change their speed-tuning classification, irrespective of stimulus mode. Most of these neurons were consistently non-speed-tuned with one neuron that was consistently speed-tuned across all three modes. Among the cells that did change classification, there were no neurons that were classified as speed-tuned in the standard mode, but became non-speed-tuned in the moving mode. Rather, many neurons that were non-speed-tuned with standard mode stimuli became speed-tuned with moving mode stimuli (52, 47.3% of the sample). Among the latter, there were 50 neurons which were also not speed-tuned when presented with stimuli in in the waxing/waning mode.

**Figure 7:**
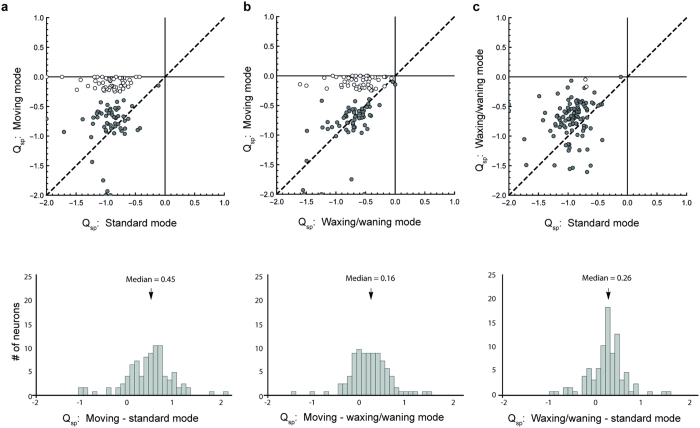
Changes in Q_sp_ between two modes. The upper row compares the values of Q_sp_ in tests conducted in (**a**) the standard and the moving modes, (**b**) the waxing/waning and the moving modes, and (**c**) the standard and the waxing/waning modes. White circles represent neurons that were changed from being non-speed-tuned (in the mode specified on the x-axis) to being speed-tuned (in the mode specified on the y-axis). Black circles represent all other neurons. The dashed lines represent values where Q_sp_ was identical in the two modes. The lower row illustrates the distributions of the difference of Q_sp_ in different modes.

We also examined how estimates of parameter Q_sp_ change for individual neurons, with respect to the stimulus presentation mode (Fig. 7, upper row). We found that, for the vast majority of neurons (93, 84.5%), changing the stimulus from the standard mode to the moving mode resulted in an increase in Q_sp_ (Fig. 7a), and that a similar marked change occurred from the waxing/waning to moving mode (Fig. 7b; 75 neurons, 68.2%). Two features about these plots were noteworthy. First, among the neurons that became speed-tuned (indicated by white dots in the scatter plots), the Q_sp_ values obtained in the comparison modes (represented in the abscissas) were widely distributed. The change was therefore not explained by a “tip of the iceberg” effect, where an equal amount of tilt in the SF-speed space was introduced by the moving stimulus, causing nearly speed-tuned neurons in the standard and waxing/waning modes to become speed-tuned. Second, there were no obvious clusters. It is therefore unlikely that the change in Q_sp_ was mediated by a mechanism that operates on specialized populations. To quantify this effect, we plotted the difference between Q_sp_ values obtained with different stimulus modes (Fig. 7, lower row). The median values were 0.45, 0.16, and 0.26 in the standard to moving, waxing/waning to moving, and standard to waxing/waning comparisons, respectively. All distributions were unimodal.

### V1 neurons show a less pronounced change in speed-tuning from drifting to moving mode

**Figure 8:**
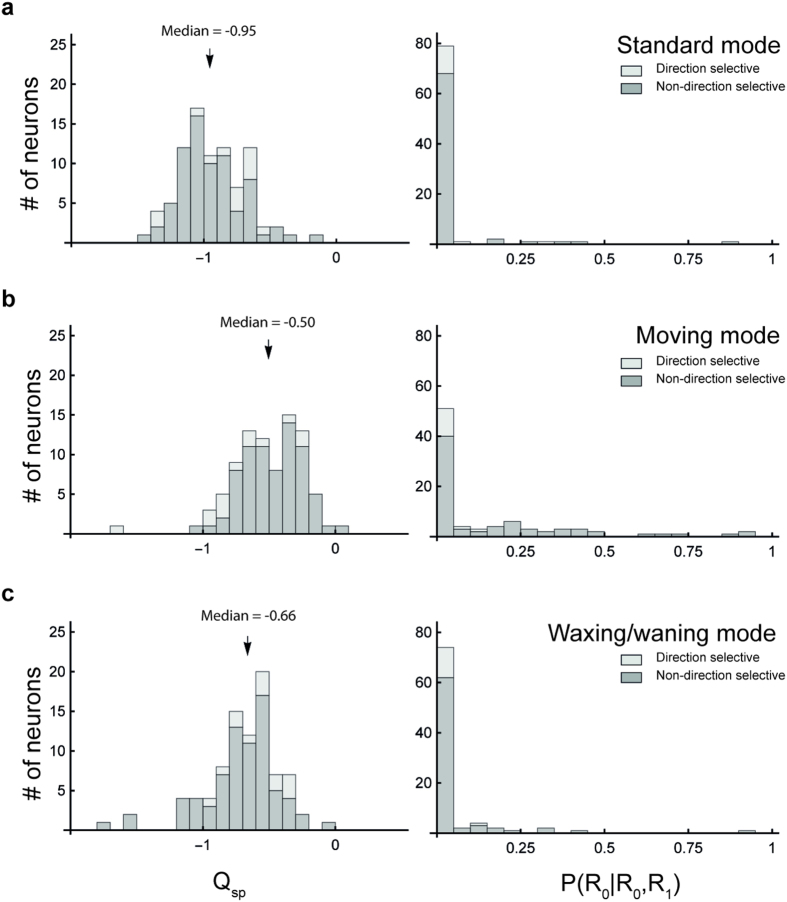
Distributions of Q_sp_ and P(*R*_0_|R_0_, *R*_1_) under the stimulation of the 3 modes in V1. The format is the same as in Fig. 6. The bars shaded by the lighter grey level represent neurons that are direction selective, whereas bars shaded by the grey level represent neurons that are not direction selective.

In order to determine whether the effect described above was a unique property of MT neurons, or inherited from V1, we also characterized speed-tuning of 87 neurons in the mid-peripheral representation of V1, using the same protocol. We found that only 7 neurons (8.0%; Fig. 8, right column) were classified as speed-tuned when stimuli were presented in the moving mode. One of these was also classified as speed-tuned in the standard mode, and two in the waxing/waning mode, according to the P(*R*_0_|*R*_0_, *R*_1_) > 0.5 criterion. The dependency between SF and speed-tuning, as indicated by the Q_sp_ parameter, decreased significantly from the median value of −0.95 in the standard mode to −0.50 in the moving mode (Mann-Whitney Test, U = 288, p < 0.0001; Fig. 8, left column). Thus, although very few V1 neurons are speed-tuned, the dependency between speed-tuning and SF tends to be reduced when stimuli were presented in moving mode. We also tested if a greater effect could be observed in an analysis restricted to a population of highly direction selective V1 neurons (n = 13) sample (indicated by bars shaded with the lighter grey level in Fig. 8); the proportion of direction-selective neurons in the sample is in agreement with earlier estimates in the marmoset^12^,^13^,^14^. However, the results of this analysis found that there were no direction selective V1 neurons classified as speed-tuned, in any mode.

## Discussion

Characterization of the responses of MT cells to moving visual patterns is widely regarded as key to our understanding of the brain mechanisms involved in visual motion processing^1^. We have addressed an important aspect of this question: to what extent can MT neurons signal the speed of a moving object in an unambiguous manner? Previous single-unit recording studies in both awake and anesthetized animals have reported that only a minority of neurons in area MT (10–25%) yielded responses that reflect tuning to the speed of the carrier wave of a grating, irrespective of its SF^4^,^5^,^6^. Refining on these studies, the present experiments used Gabor patches as visual stimuli, instead of sharply delimited gratings, to avoid the possibility of inadvertent stimulation of the neuron’s receptive field by other spatial and temporal frequencies (which could affect estimates of speed-tuning^4^). In tests where Gabor patches were presented in the standard (drifting) mode, we found an even lower proportion (1-2%) of speed-tuned cells in MT, in comparison with previous work using traditional gratings (including a study that used otherwise identical methods^5^). However, our key observation here is that many of these same cells, amounting to approximately half of the entire MT population, revealed SF-invariant speed tuning in tests where the Gabor patch moved along a trajectory that crossed the receptive field. Our results suggest a form of spatiotemporal contextual effect, in which the prior history of the stimulus influences how information is processed in MT.

Many computational models of motion processing in MT^8^,^9^,^15^,^16^,^17^ incorporate nonlinear elements such as static linearity and divisive normalisation, but integrate activities across spatiotemporal channels linearly. Under the linear pooling assumption, a neuron’s response profile to the speed of a moving stimulus would be predictable from its responses to drifting gratings, despite the nonlinear elements in the model. The fact that approximately half of the neurons in our sample changed classification, from non-speed-tuned in the standard mode to speed-tuned in the moving mode, indicates significant nonlinear interactions among spatiotemporal channels in a large population of MT neurons. Psychophysical experiments using stimuli consisting of the sum of two gratings have reported nonlinear summation behaviour in speed judgments^18^. In addition, Priebe *et al.* (2003) superimposed two drifting gratings with different SFs but the same speed, and reported that responses of neurons in macaque MT were more tuned to speed of the superimposed gratings than the predicted responses patterns as measured by single drifting gratings^4^. Our findings are compatible with Priebe *et al.* (2003), because the moving mode stimulus contained a richer set of spatiotemporal frequencies, compatible with the spatial displacement of the envelope of the Gabor pattern in a trajectory (Fig. 2). Importantly, we further showed that although nonlinear summation can alter how motion signals are processed in MT, the effect was observed only in about half of the population. Thus, TF-based computation is still an important component in the cortical processing of motion signals.

The novel aspect of the moving mode stimulus is that the Gabor pattern travelled across the receptive field. An interesting possibility is that the temporal context of the moving trajectory allows the history of the stimulus outside the receptive field to influence how information is processed when the stimulus is in the receptive field. Such mechanisms have been suggested by psychophysical studies^19^,^20^,^21^,^22^. Further studies are required to test this possibility.

In addition to the moving mode, we also used a variant of drifting stimuli (waxing/waning mode) in which the duration of presentation of the Gabor patch was consistent with that of a moving mode stimulus through an aperture. The varying duration (and therefore the varying TF bandwidth) of the stimulus in this presentation mode was found to have no significant effect on the proportion of speed-tuned neurons in MT, in comparison to the standard mode. This result suggests that dependence on the temporal properties of a stimulus is not marked, in terms of determining the degree of speed-tuning. However, the fact that the vast majority of the neurons in our sample only showed robust speed-tuning when stimuli were presented in the moving mode is consistent with the idea that the visual system has evolved to be most efficient in encoding naturally occurring features^23^,^24^. This idea has been tested by Nishimoto & Gallant (2011)^17^, who studied the receptive field structure of MT neurons in awake macaques viewing motion-enhanced natural movies. In that study, three-dimensional spectral receptive fields were estimated by fitting the responses to a multi-filter model with static nonlinearity for individual filter outputs, which were then normalised and linearly summed. A continuum of speed selectivity was found, with a significant population of neurons showing speed-tuning. However, since the 3-dimensional plan fitting procedure used for quantifying speed-tuning in Nishimoto and Gallant (2011) is quite different from both the Q_sp_ parameter used in the present study (and analogous estimates in other previous studies), it is difficult to directly compare the proportion of speed-tuned neurons. Our results show that the shapes of MT spectral receptive fields are dynamic, and can be changed from being non-speed tuned to speed-tuned by a manipulation as simple as displacing the Gabor pattern on a trajectory in time.

The data shown in Fig. 7 indicate that there are incremental changes in the dependency between the SF of the stimulus and the optimal speed across the entire dataset, rather than suggesting the existence of a specific population of MT cells. Contextual effects associated with motion trajectories in the moving mode might be mediated by propagation of signals via intrinsic horizontal connections in MT^25^,^26^ or by feedback circuits originated from higher order visual areas, such as the medial superior temporal (MST) or the lateral intraparietal (LIP) areas.

Area MT has been considered the central node of the cortical network involved in motion processing. Determining if MT neurons’ computational properties are unique to the area, or if they are inherited from computations performed in V1 is important for understanding the architecture of information processing in the visual cortex^27^. The importance of interactions between V1 and MT has also been highlighted in an earlier study that demonstrated that MT neurons fail to develop direction selectivity when V1 is lesioned in early postnatal life^28^. Our moving mode stimulus revealed very few (7; 8.0%) speed-tuned V1 neurons, with 4 showing speed-tuning only when the stimulus was the moving mode. The result is somewhat inconsistent with the findings of Priebe *et al.* (2006)^29^, who showed a larger population (~25%) of speed-tuned cells in macaque monkey’s V1, but minimal nonlinear interactions when two drifting gratings were combined. Although both studies suggest a degree of functional overlap between neurons in V1 and in MT, they also indicate that MT neurons perform a distinct set of computations, which enable accurate perception of speed as well as direction of motion, in adult primates^30^.

## Methods

Single-unit recordings were obtained from the primary visual cortex (V1) and area MT in 6 adult New World monkeys (*Callithrix jacchus*, the common marmoset). Apart from one animal in which both areas were tested, recordings were performed in either MT (n = 3) or V1 (n = 2). Experiments were conducted in accordance with the Australian Code of Practice for the Care and Use of Animals for Scientific Purposes, and all procedures were approved by the Monash University Animal Ethics Experimentation Committee.

### Animal preparation

Preparation of the animal for *in vivo* recordings of neurons was based on the protocol described in detail by Bourne and Rosa (2003)^31^, and updated according to Yu *et al.* (2010)^11^. Anaesthesia was induced with alfaxalone (10 mg kg^−1^), allowing a tracheotomy, a vein cannulation and a craniotomy to be performed. After all surgical procedures were completed, the animal was constantly administered an intravenous infusion of pancuronium bromide (0.1 mg kg^−1^ h^−1^) combined with sufentanil (8 μg kg^−1 ^h^−1^) and dexamethasone (0.4 mg kg^−1^ h^−1^). In addition, it was artificially ventilated with a gaseous mixture of N_2_O and O_2_ (7:3). The electrocardiogram, peripheral capillary oxygen saturation and level of spontaneous activity in the cortex were continuously monitored. Administration of atropine (1%) and phenylephrine hydrochloride (10%) eye drops resulted in mydriasis and cycloplegia. Appropriate focus and protection of the cornea from desiccation were achieved using contact lenses, which brought into focus the surface of a calibrated CRT monitor (Sony Multiscan G520, 100 Hz refresh rate) located 30–50 cm from the animal. The width of the monitor covered 40°–60° of the visual field. Visual stimuli were presented to the eye contralateral to the hemisphere from which the neuronal recordings were obtained.

### Electrophysiological recordings

Parylene-coated tungsten microelectrodes (~1 MΩ), with exposed tips of 10 μm were directed towards areas V1 or MT based on stereotaxic coordinates^32^. The location of the electrode in these areas was confirmed using visual topography and, in the case of MT, the presence of direction-selective neurons^10^. The electrode was slowly advanced into the cortex through a small slit in the dura mater, until the first units could be observed above background activity. The data were collected using Expo software (designed by Peter Lennie and others), which also allowed for online spike discrimination.

### Experimental protocol

Within each electrode penetration, neurons were sampled at 100–300 μm intervals. At each location, the boundary of the multiunit minimum response receptive field was carefully mapped using a manually operated stimulus (high-contrast moving bars, small grating patches and flashing light spots) generated on the computer monitor, while the response was monitored through a loudspeaker. Following delineation of the receptive field boundaries, the activity of the largest unit (or units) responding reliably to visual stimulation was isolated while a drifting grating was displayed on the screen, and if necessary, estimates of receptive field boundaries were adjusted.

The direction tuning curve of the isolated unit was obtained using a drifting sinusoidal grating, centred on the receptive field, whose SF and TF were adjusted in order to elicit strong responses. For this test the grating drifted for 2 s in one of 12 directions (30^o^ steps), which were presented in a random sequence within each block.

After the optimal direction of motion of a neuron was determined, we conducted three series of tests, corresponding to the three stimulus modes described in the Results section (see Supplementary Video 1 for a demonstrating the three modes). These tests were designed to quantify the interaction between responses to different speeds, using stimuli of different SF. In all three tests, high contrast (80%) Gabor patterns with independently varying SF and speed were presented in a 5 × 5 matrix of conditions (e.g. Fig. 3). The ranges of both parameters were adjusted for each neuron, based on the results of preliminary tests, to ensure that the dynamic ranges were adequately sampled. An additional “blank” condition was included, where a grey screen of average luminance between the high and low luminance peaks of the Gabor pattern was displayed for 2 s, to measure spontaneous activity. Each condition was repeated 8 times.

In the three tests, Gabor patterns were presented in three different modes (Fig. 1):

- *Standard mode*: In this mode, a Gabor patch appeared over the receptive field as a static pattern for 1 s. The carrier wave then drifted for 2 s at a constant speed, in the optimal direction of the neuron. The neuron’s level of stimulus-evoked activity was measured during the constant motion period, to avoid the transient response evoked by its onset.
- *Moving mode*: In this mode, a Gabor patch appeared well outside the boundary of the receptive field, remained as a static pattern for 1 s, and then moved at a constant speed towards and across the receptive field, in the optimal direction for the neuron. In this stimulus mode, the Gabor envelope changed its spatial location, while the carrier wave remained stationary with respect to the envelope.
- *Waxing/waning mode*: Similar to the standard mode, the stimulus envelope remained stationary relative to the receptive field, while the carrier wave drifted at a constant speed. However, the global contrast of the Gabor patch was modulated in such a way that the time course of the modulation of the stimulus at the centre of the receptive field was matched to that of a corresponding condition in the moving mode.

### Data analysis

For each SF-speed combination in the three modes, we calculated space-equalized peri-stimulus time histograms (PSTHs) by converting spike times to a spatial scale, which represented the distance travelled by a fixed point in the stimulus (according to *s* = v*t, where *s* is the spatial distance travelled, *t* is the time spike and *v* is the stimulus speed; see Supplementary Figure S1). The bin sizes were adjusted according to the speed of the stimulus. They were chosen such that each bin represented a time interval during which the Gabor pattern travelled a constant distance in the moving mode.

To estimate the mean evoked response rate for each condition, we calculated the number of action potentials that occurred within a defined window in the space-equalised PSTH (see the shaded regions in the PSTHs shown in Figs 3 and 5). For the standard mode, a fixed window was used for all 25 SF-speed combinations. The window was determined by first collapsing all 25 conditions into a space-equalized grand PSTH, which was then fitted to a log-transformed skewed-Gaussian function


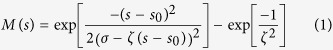


where 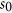 is the point of peak response, 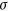 represents the width, and 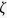 the skewness of the fitted curve. The window was set to (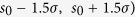.

For the moving mode, the windows were chosen to capture the responses in the time intervals during which the Gabor pattern was inside the receptive field. Since the time course of the stimulus was determined by the speed of the moving Gabor pattern, the same window (in terms of its width and location) was used for all conditions in which the Gabor pattern had the same speed but different SF. Moreover, the 5 windows had the same width on the space-equalised PSTHs (i.e., they represented the same travel distance), but the onsets were allowed to vary to accommodate variations of the response dynamics due to the speed of the Gabor pattern (see Supplementary Figure S1). To calculate the windows, conditions with the same speed but different SF were collapsed to form 5 space-equalised PSTHs, which were then fitted individually to skewed-Gaussian functions. For each speed, the (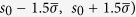 window was used, where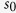 represents the location of the peak response, and 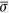 represents the mean value of the 5 fitted 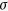 parameters.

For the waxing/waning mode, the mean response rates were calculated using spikes that occurred in the time intervals during which the contrast of the Gabor pattern was greater than 0. Since the time course of the waxing/waning mode was matched to that of the moving mode (Fig. 1), the same procedure used for the moving mode was used. For each isolated unit, the windows for the moving and the waxing/waning mode were obtained separately, to accommodate potential differences in response latency in the two modes.

The mean spike rates, calculated within the window, produced a tuning surface that represented the neuronal responses as a function of speed and SF (e.g. Figs 3 and 5, third column).

The tuning surface obtained for each unit was fitted with two competing models^11^: the SF-speed separable model (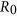) and the SF-speed inseparable model (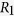). In the SF-speed separable model (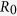), the SF and speed-tuning curves (denoted as 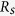 and 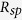 respectively in Eq. 2) are assumed to be independent of each other. Consequently the spatiotemporal tuning surface 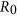 is the product of the two:


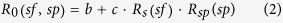


In this formulation, both 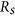 and 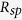 are modeled as skewed normal functions in logarithmic space:


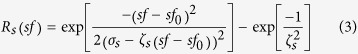



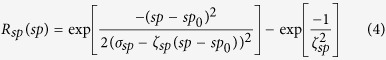


where 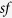 and 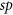 are SF and speed transformed by log_2_. Therefore, 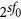 and 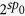 are the optimal SF and speed. 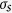 and 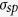 are the widths of the tuning curves, and 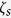 and 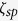 are skewness factors. There are eight free parameters in the separable model.

The SF-speed inseparable model (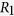) involves an extra parameter 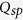 to characterize the interaction between the spatial and temporal domains. It is expressed as:


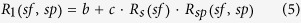


where 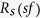 is the same as in Eq. (3), and


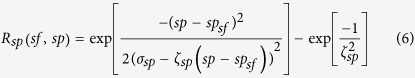



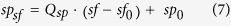


When 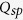 is 0, 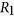 is equivalent to the SF-speed separable model 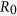 (Fig. 4c). The interpretation of 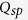
is illustrated in Fig. 4.

The 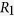 model is similar to those used in previous studies^4^,^5^,^6^,^11^,^29^,^33^,^34^, except that in those studies, models were expressed in the SF-TF space, rather than in SF-speed space. The 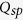 parameter in the present study is analogous to the 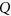 parameter used in the previous studies. The models were fitted to the spike rate of every trial, rather than the mean spike rate of each condition.

The Bayesian Information Criterion (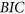) was used to assess whether the SF-speed separable model (R_0_) was sufficient to encapsulate our observations on a given neuron’s responses. This method determined if 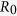 provided a better fit of the data, when model complexity was taken into account. Formally, 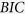 is in inverse proportion to the posterior probability of the observed data given a model, and is expressed as 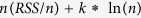, where *n* is the number of data points, *k* is the number of free parameters in the model and 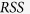
is the residual sum of squares from the estimated model. The probability that the data are better explained by 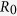, rather than 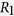, is determined by


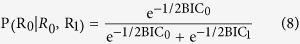


where 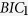 is the 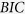 of the SF-speed inseparable model, and 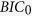
is the 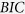 of the SF-speed separable model.

### Histology

The electrode tracks were made more readily visible with the aid of small electrolytic lesions (4 μA, 15s), which were placed at various sites during the experiment. At the end of the experiments, the animals were administered an overdose of sodium pentobarbitone and perfused transcardially with 0.9% saline, followed by 4% paraformaldehyde in 0.1M phosphate buffer (pH 7.4). After cryoprotection by increasing concentrations of sucrose, and sectioning, alternate slides (40 μm) were stained for Nissl substance with cresyl violet, and for myelin^35^, allowing reconstruction of electrode tracks and electrolytic lesions relative to histological borders. Area MT was identified by its heavy myelination^10^. In one animal an alternate series of sections was stained for cytochrome oxidase^36^, which also reveals the boundaries of MT^28^. Electrode tracks in V1 were identified using well-established criteria, such as the presence of the stria of Gennari.

## Additional Information

**How to cite this article**: Davies, A. J. *et al.* Natural motion trajectory enhances the coding of speed in primate extrastriate cortex. *Sci. Rep.* **6**, 19739; doi: 10.1038/srep19739 (2016).

## Supplementary Material

Supplementary Video 1

Supplementary Information

## Figures and Tables

**Table 1:** The numbers of MT neurons in all possible speed-tuned/non speed-tuned combinations for the three modes.

Standard mode	Moving mode	Waxing/waning mode	Number
Speed- tuned	Speed- tuned	Speed-tuned	1 (0.9%)
Speed- tuned	Non speed- tuned	Speed- tuned	0 (0%)
Speed- tuned	Speed- tuned	Non speed- tuned	0 (0%)
Speed- tuned	Non speed- tuned	Non speed- tuned	0 (0%)
Non speed- tuned	Speed- tuned	Speed- tuned	2 (1.8%)
Non speed- tuned	Non speed- tuned	Speed- tuned	0 (0%)
Non speed- tuned	Speed- tuned	Non speed- tuned	50 (45.5%)
Non speed- tuned	Non speed- tuned	Non speed- tuned	57 (51.8%)

The classification was based on the P(*R*_0_|*R*_0_, *R*_1_) >0.5 criterion.
