# Supplementary figures and images for: Natural motion trajectory enhances the coding of speed in primate extrastriate cortex
Source: Sci Rep. 2016 Jan 27;6:19739. doi: 10.1038/srep19739 (PMC4728434; doi:10.1038/srep19739)

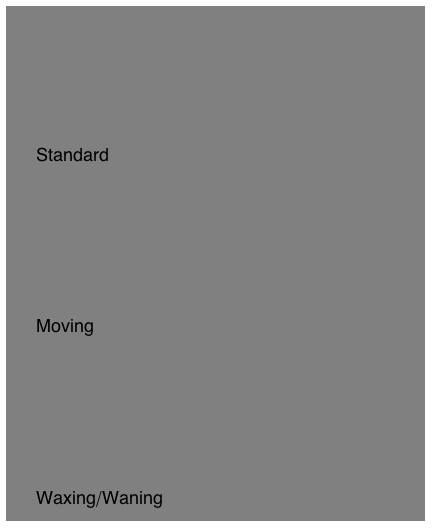

Supplement: Supplementary Video 1 [file srep19739-s1.gif]
