# Supplementary material for: Natural motion trajectory enhances the coding of speed in primate extrastriate cortex
Source: Sci Rep. 2016 Jan 27;6:19739. doi: 10.1038/srep19739 (PMC4728434; doi:10.1038/srep19739)

Supplementary Information

Natural motion trajectory enhances the coding of speed in primate extrastriate cortex

Amanda J. Davies, Tristan A. Chaplin, Marcello G.P. Rosa and Hsin-Hao Yu

Contents:

Supplementary Figure S1

Supplementary Video 1 (in animated GIF format, Davies_et_al_Video1.gif)

Supplementary Figure S1: An illustration of the calculation of the space-equalized PSTH for the moving mode. In (**a**), the PSTHs of a Gabor pattern (central SF=0.39 c/°) moving at three different speeds are plotted. The PSTHs were converted to space-equalized PSTHs in (**b**). The sizes of the bins were adjusted such that the distances travelled by the Gabor pattern were the same in the duration of each bin. The red curves indicate the skewed Gaussian functions fitted to the space-equalized PSTHs, and the shaded regions indicate the windows used to calculate the mean response rates. The windows had the same widths (number of bins) but the positions were allowed to vary to allow for variations in response latency.

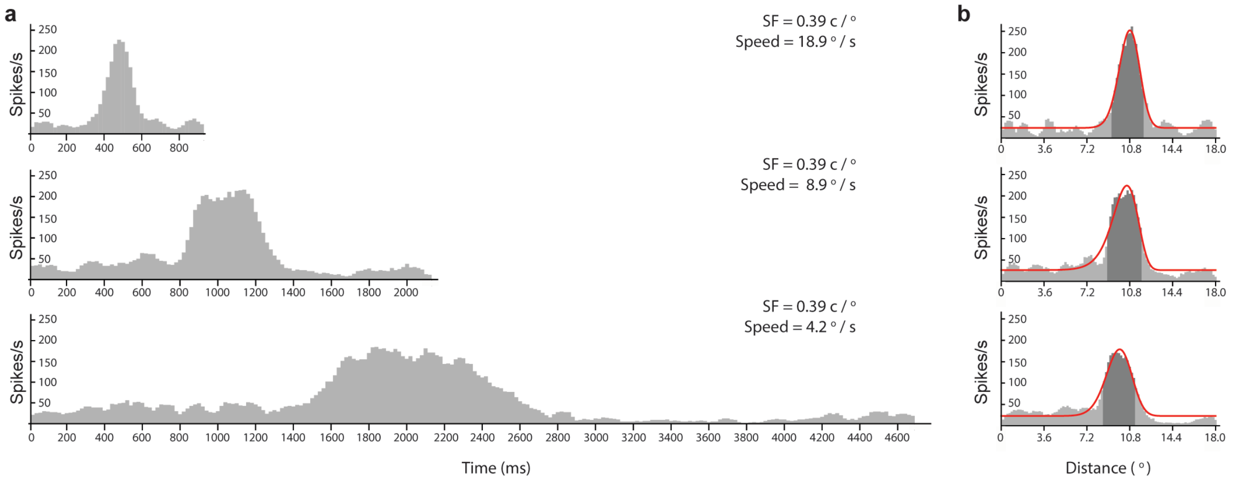

Supplementary Video 1: An animation that illustrates the three stimulus presentation modes.

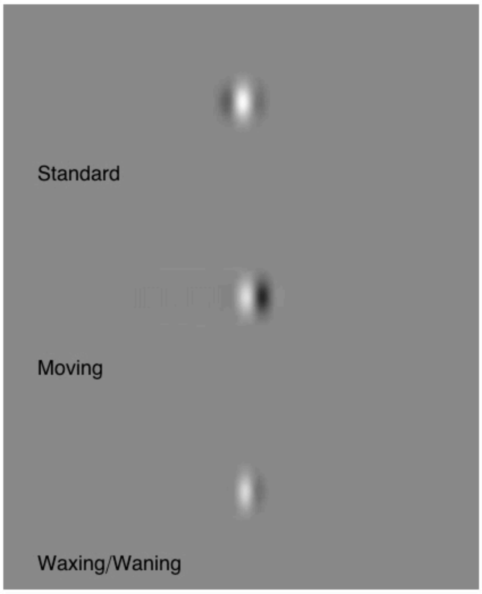

Supplement: Supplementary Information [file srep19739-s2.pdf]
